# The complete mitochondrial genome of the smudged eighty-eight butterfly *Diaethria gabaza eupepla* (Salvin & Godman, 1868) (Insecta: Lepidoptera: Nymphalidae)

**DOI:** 10.1080/23802359.2022.2065220

**Published:** 2022-04-20

**Affiliations:** Department of Biological Sciences, University of Manitoba, Winnipeg, MB, Canada

**Keywords:** Illumina sequencing, mitogenomics*;* Lepidoptera, Nymphalidae, *Diaethria*

## Abstract

The smudged eighty-eight butterfly *Diaethria gabaza eupepla* (Salvin & Godman, 1868) (Nymphalidae) is a vividly colored aposematic butterfly from Central and South America. A complete circular mitochondrial genome (mitogenome) of 15,156 bp from *D. gabaza eupepla* was assembled from a genome skimming Illumina sequence library. The AT-rich (80.5% AT) mitogenome consists of 13 protein-coding genes, 22 tRNAs, 2 rRNAs, and a control region in the typical butterfly gene order. *Diaethria gabaza eupepla COX1* begins with an atypical CGA start codon and *ATP6, COX1*, *COX2, CYTB, ND1, ND4, ND4L*, and *ND5* mRNAs contain incomplete stop codons completed by the addition of 3’ A residues. Phylogenetic reconstruction places *Diaethria* as the sister clade to *Hamadryas* within monophyletic nymphalid subfamily Biblidinae, consistent with previous phylogenetic hypotheses.

The Living Prairie Mitogenomics Consortium is a structured inquiry exercise for undergraduates (Marcus et al. 2010) who assemble arthropod mitogenomes for improved DNA-based species identification and phylogenetics (Living Prairie Mitogenomics Consortium 2017, 2018, 2019, 2020, 2021). Participating students assembled, annotated, and analyzed sequence data (further curated by the instructor) and conducted a literature review for presentation here.

The butterflies of the genus *Diaethria* Billberg, 1820 (Nymphalidae: Biblidinae: Callicorini) are notable in that they possess color patterns on the ventral hind wings that resemble the numerals “88” or “89” and so are known by the common name “eighty-eight butterflies” (Dias et al. 2012). These distinctive patterns create a black, white, and red aposematic wing display intended to deter predators from eating these butterflies (Chai 1986; Pinheiro 1996). The genus includes a dozen species that occur between Texas, USA and Tierra del Fuego, Argentina (Dias et al. 2012). Male *Diaethria* are strongly attracted to urine-soaked sand and engage in mud-puddling behavior (DeVries 1987). The smudged eighty-eight butterfly *Diaethria gabaza* (Hewitson 1855) includes 3 subspecies, with subspecies *D. gabaza eupepla* (Salvin & Godman, 1868) occurring between Costa Rica and Colombia, where its larvae feed on climbing vines in the genus *Serjania* (Sapindaceae) (DeVries 1987).

Here we report the complete mitochondrial genome (mitogenome) sequence of *D. gabaza eupepla* from specimen Diae2019.1, collected by John R. MacDonald on 6 October 2019 at Finca Hartmann, Panama (GPS 8.844943 N, 82.760747 W) that has been pinned, spread, and deposited in the Wallis Roughley Museum of Entomology, University of Manitoba (http://www.wallisroughley.ca/, Jason Gibbs, Jason.Gibbs@umanitoba.ca) under the voucher number WRME0507740. This study was conducted with the approval of the University of Manitoba Office of Research Ethics & Compliance under permit number BF0155-1. Research was carried out in accordance with applicable national and international guidelines.

A leg was removed from the specimen and DNA was prepared using a DNEasy Blood and Tissue kit (Qiagen, Düsseldorf, Germany) with slight protocol modifications as described in McCullagh and Marcus (2015). DNA was sheared by sonication and a fragment library was prepared as previously described (Peters and Marcus 2017) using a NEBNext Ultra II DNA Library Prep Kit before sequencing by Illumina NovaSeq6000 (San Diego, CA) (Marcus 2018). Mitogenome assembly and annotation of the *D. gabaza eupepla* (GenBank accession MZ981736) was performed by mapping the resulting sequence library of 57,341,531 paired 150 bp reads (GenBank SRA PRJNA759138) to a *Baeotus beotus* reference mitogenome (Lepidoptera: Nymphalidae: Nympahlina: Coeini, MW566598 (Lalonde 2021)) using 5 iterations of the medium sensitivity settings of Geneious Prime 2021.1.1. Nuclear rRNA repeat sequences are increasingly recognized as being very useful for phylogenetic comparisons (Dodsworth 2015; Coissac et al. 2016; Marcus 2018; Krehenwinkel et al. 2019), so we also assembled the complete *D. gabaza eupepla* nuclear rRNA repeat (GenBank MZ981737) using a *B. beotus* (MW571038) (Lalonde 2021) reference sequence.

The *D. gabaza eupepla* circular 15,156 bp mitogenome assembly was composed of 22,600 paired reads with nucleotide composition: 39.8% A, 11.7% C, 7.8% G, and 40.7% T. The gene composition and order in *D. gabaza eupepla* is typical of the arrangement found in most butterfly mitogenomes (Park et al. 2016). The protein-coding genes start codons include: ATG (*ATP6*, *COX2*, *COX3*, *CYTB*, *ND1*, *ND4*), ATT (*ND2*, *ND3*, *ND6*), ATA (*ND4L, ND5*), and ATC (*ATP8*), while *COX1* begins with an atypical CGA start codon as in many other insects (Liao et al. 2010). The mitogenome contains four protein-coding genes (*COX1*, *COX2, ND4, ND5*) with single-nucleotide (T) stop codons, and four protein-coding genes (*ATP6, CYTB, ND1, ND4L*) with two-nucleotide (TA) stop codons completed by post-transcriptional addition of 3′ A residues. The locations and structures of tRNAs were determined using ARWEN v.1.2 (Laslett and Canback 2008). All tRNAs exhibited cloverleaf secondary structure except that the dihydrouridine arm of trnS (AGN) was replaced by a loop. The size and structure of the mitochondrial rRNAs and control region are typical for Lepidoptera (McCullagh and Marcus 2015).

Phylogenetic reconstruction (Figure 1) was conducted using the complete mitogenome of *D. gabaza eupepla* and 31 other complete mitogenomes from the family Nymphalidae, including outgroup species *Limenitis sydyi* from nymphalid subfamily Limenitidinae and available from GenBank (Alexiuk et al. 2020; Hamilton et al. 2020; Lalonde and Marcus 2020; Payment et al. 2020; Alexiuk et al. 2021a, 2021b; Lalonde 2021). GenBank accession numbers are listed in Figure 1. Mitogenome sequences were aligned in CLUSTAL Omega (Sievers et al. 2011) and analyzed using Bayesian Inference with the GTR + I + G model (model selected using jModeltest 2.1.1 (Darriba et al. 2012)) in Mr. Bayes version 3.2.7 (Ronquist and Huelsenbeck 2003; Ronquist et al. 2012). As expected based on a previous phylogenetic hypothesis (Wahlberg et al. 2009), phylogenetic analysis placed *D. gabaza eupepla* as the sister taxon to *Hamadryas epinome* in a monophyletic clade with mitogenomes from nymphalid subfamily Biblidinae.

**Figure 1.**
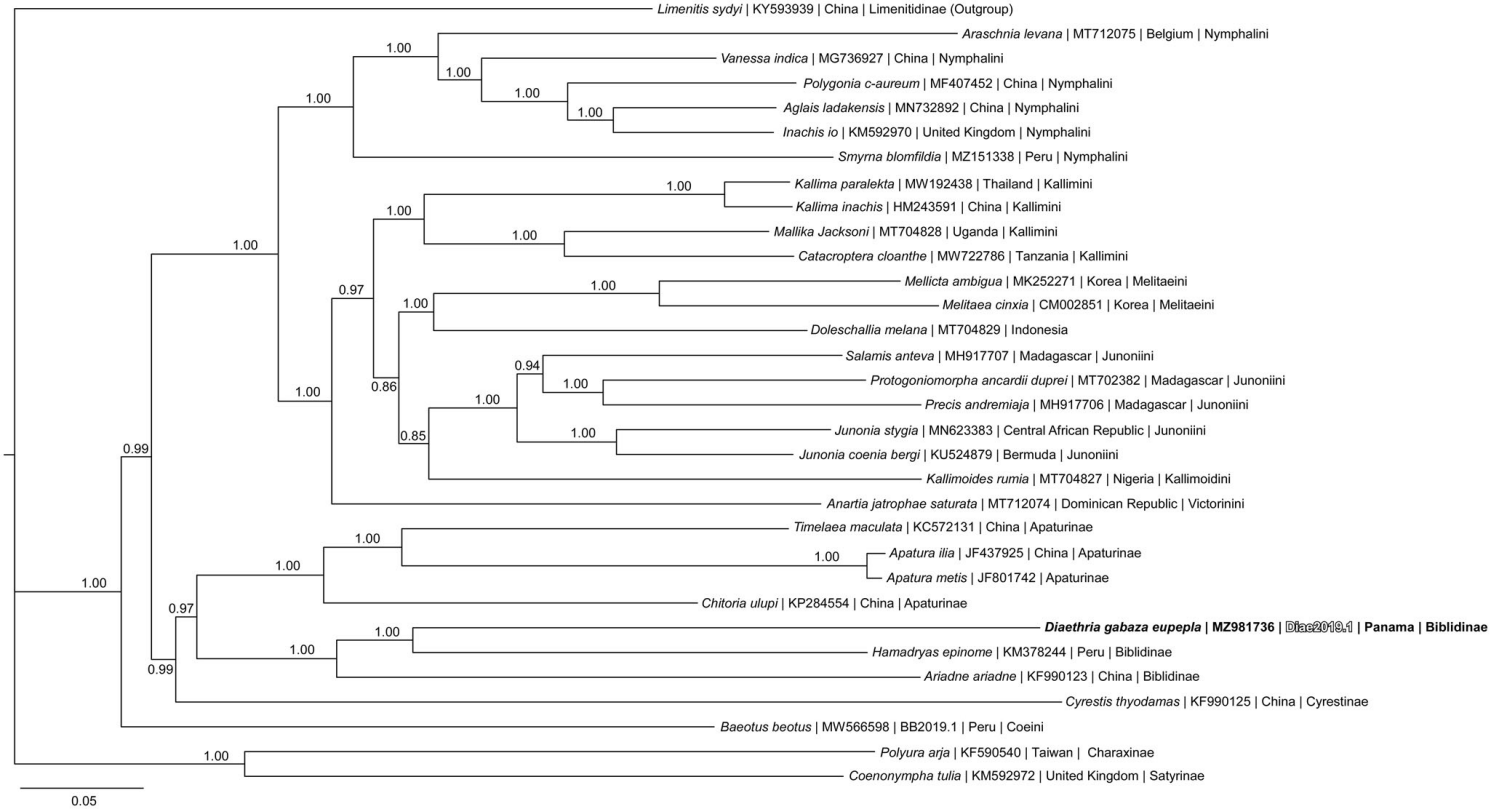
The Bayesian phylogeny (GTR + I + G model, best state likelihood = −147,240.16, average deviation of split frequencies = 0.001131) of the *Diaethria gabaza eupepla* mitogenome, 31 additional mitogenomes from within family Nymphalidae, including outgroup species *Limenitis sydyi* (Nymphalidae: Limenitinae), produced by 10 million MCMC generations in MrBayes, with sampling every 1000 generations, and after discarding the first 250,000 generations as burn-in. The Bayesian posterior probability values determined by Mr Bayes are provided at each node. Each taxon in the analysis is labeled with species name, GenBank accession, the country of origin of the specimen with the sequenced mitogenome, and the nymphalid Tribe or Subfamily of the species.

## Data Availability

The genome sequence data that support the findings of this study are openly available in GenBank of NCBI at [https://www.ncbi.nlm.nih.gov] (https://www.ncbi.nlm.nih.gov/) under the accession no. MZ981736–MZ981737. The associated BioProject, SRA, and Bio-Sample numbers are PRJNA759138, SRX11982685, and SAMN21156917 respectively.
